# *Trichoderma reesei* FS10-C enhances phytoremediation of Cd-contaminated soil by *Sedum plumbizincicola* and associated soil microbial activities

**DOI:** 10.3389/fpls.2015.00438

**Published:** 2015-06-10

**Authors:** Ying Teng, Yang Luo, Wenting Ma, Lingjia Zhu, Wenjie Ren, Yongming Luo, Peter Christie, Zhengao Li

**Affiliations:** Key Laboratory of Soil Environment and Pollution Remediation, Institute of Soil Science, Chinese Academy of SciencesNanjing, China

**Keywords:** *Trichoderma reesei*, FS10-C, cadmium, phytoremediation, *Sedum plumbizincicola*, soil microbial activities

## Abstract

This study aimed to explore the effects of *Trichoderma reesei* FS10-C on the phytoremediation of Cd-contaminated soil by the hyperaccumulator *Sedum plumbizincicola* and on soil fertility. The Cd tolerance of *T. reesei* FS10-C was characterized and then a pot experiment was conducted to investigate the growth and Cd uptake of *S. plumbizincicola* with the addition of inoculation agents in the presence and absence of *T. reesei* FS10-C. The results indicated that FS10-C possessed high Cd resistance (up to 300 mg L^-1^). All inoculation agents investigated enhanced plant shoot biomass by 6–53% of fresh weight and 16–61% of dry weight and Cd uptake by the shoots by 10–53% compared with the control. All inoculation agents also played critical roles in increasing soil microbial biomass and microbial activities (such as biomass C, dehydrogenase activity and fluorescein diacetate hydrolysis activity). Two inoculation agents accompanied by FS10-C were also superior to the inoculation agents, indicating that *T. reesei* FS10-C was effective in enhancing both Cd phytoremediation by *S. plumbizincicola* and soil fertility. Furthermore, solid fermentation powder of FS10-C showed the greatest capacity to enhance plant growth, Cd uptake, nutrient release, microbial biomass and activities, as indicated by its superior ability to promote colonization by *Trichoderma*. The solid fermentation powder of FS10-C might serve as a suitable inoculation agent for *T. reesei* FS10-C to enhance both the phytoremediation efficiency of Cd-contaminated soil and soil fertility.

## Introduction

Soil contamination by heavy metals (HMs) such as cadmium released from agricultural and industrial activities is an environmental problem worldwide. Potentially toxic HMs are resistant to biodegradation and their persistence thus threatens the environment and public health (Lima et al., 2011; Sahu et al., 2012). A number of methods have been developed for the remediation of metal-contaminated soils and phytoremediation is considered to be a promising technique because it is cost-effective and environmentally friendly. However, its use in field conditions has been somewhat restricted (Li et al., 2011; Shin et al., 2012; Wu et al., 2012).

In recent years plant-associated bacteria and fungi have been examined for their capacity to enhance the efficiency of phytoremediation (Rajkumar et al., 2012). Numerous filamentous fungi such as *Trichoderma* sp. have aroused increasing interest due to their potential for enhancing the establishment of vegetation and the remediation of metal-contaminated soils (Bareen et al., 2012). Moreover, *Trichoderma* species are characterized by rapid growth, asexual reproduction, effective colonization capacity and low-specificity plant symbiosis (Williams et al., 2003; Harman et al., 2004; Zafar et al., 2007). Certain *Trichoderma* species have been reported to enhance plant growth and metal availability to plants in contaminated soils. Babu et al. (2014b) reported that *Trichoderma virens* PDR-28 increased the dry biomass of maize and its Cd accumulation compared with the control. Similarly, *T. pseudokoningii* increased the biomass and Cd accumulation of pearl millet (Bareen et al., 2012). However, little is known about the effects of *T. reesei* on the phytoremediation of HM contaminated soils.

The objectives of the present study were to explore the effects of *Trichoderma reesei* FS10-C (isolated and preserved previously in our laboratory) on Cd phytoremediation by *Sedum plumbizincicola*, a Cd hyperaccumulator (Wu et al., 2012; Hu et al., 2013), to evaluate soil fertility after phytoremediation, mainly based on microbial biomass and activities, and to determine the colonization ability of *Trichoderma*.

## Materials and Methods

### Cd Tolerance and Morphological Analysis of *Trichoderma reesei* FS10-C

The Cd tolerance of FS10-C was examined by incubation at 28°C on Potato Dextrose agar (PDA; Tapia et al., 2011) and in corresponding liquid media containing 0, 5, 10, 15, 50, 100, 150, 200, 250, and 300 mg L^-1^ Cd^2+^ (CdCl_2_⋅2.5H_2_O). Colony diameters were measured after growth for 3 days. Mycelia in the conical flasks were collected after 5 days and then dried at 70°C for 24 h before being weighed. Furthermore, the EC_50_ of FS10-C under Cd stress was calculated by the linear interpolation method (Hughes et al., 2001).

The morphological changes in FS10-C under Cd stress were studied by incubating the activated strain in Potato Dextrose (PD) media at 28°C and at 150 rev min^-1^ for 5 days. Equal amounts of mycelia were picked off and added to Czapek’s medium (Kexiang et al., 2002) spiked with 0, 10, and 100 mg L^-1^ Cd^2+^ (CdCl_2_⋅2.5H_2_O) respectively for further incubation for 15 h at 28°C and at 150 rev min^-1^. A moderate amount of mycelium was then extracted, immobilized with 4% glutaraldehyde solution prepared using 0.2 mol L^-1^ phosphate buffered saline (PBS, pH 7.2) for 4 h, and washed with 0.1 mol L^-1^ PBS (pH 7.2) three times. After ethanol dehydration, the ethanol was replaced twice with isoamylacetate, for 15 min each time. Finally, electrical conductivity (15 mA, 90 s) was measured after critical point drying. Treated samples were then observed with a scanning electron microscope (SEM, FEI Quanta 200, Hillsboro, OR, USA) and evaluated qualitatively and quantitatively with an Energy Dispersive X-ray Detector (EDX, INCA E-250, High Wycombe, UK).

### Preparation of Inoculation Agents

To apply *Trichoderma* sp. widely in practice the first task is to obtain a large number of *Trichoderma* products. Thus far, the *Trichoderma* agents produced in commercial applications have been intended primarily for spore preparation. In this context, we prepared several inoculation agents using the following preparation methods. The fermentation conditions and proportion parameters were explored in orthogonal experiments.

*Trichoderma reesei* FS10-C was first activated in PDA media and then prepared as a spore suspension (1 × 10^6^ colony-forming units mL^-1^). The spore suspension was inoculated onto a sterilized solid matrix (1:20 v/w) and incubated at 28°C for 10 days. The solid matrix consisted of orange peel powder and wheat bran (1:1 w/w) with the moisture content adjusted to 50% w/v using deionized water. This fermented product was designated ‘solid fermentation powder of *T. reesei* FS10-C’ and its efficacy was examined with and without sterilization (the first and second inoculation agents).

Conidium wettable powder of *T. reesei* FS10-C was the third inoculation agent, consisting of a mixture of 10% *T. reesei* FS10-C conidium powder, 5% sodium dodecyl benzene sulfonic acid, 0.4% vitamin C, 10% kaolinite and 74.6% sodium lignosulphonate. This mixture, without the addition of 10% *T. reesei* FS10-C conidium powder, was used as the fourth inoculation agent. These latter two inoculation agents were diluted 500 times before use.

### Sample Collection and Experimental Design

Soil samples were collected from the arable layer (top 15 cm) of a Cd-contaminated agricultural soil located in Xiangtan, Hunan Province, China. The physicochemical properties and Cd content of the soil samples are shown in **Table 1**. The soil was air dried, sieved (2 mm), and mixed thoroughly with 0.15 g kg^-1^ N as (NH_4_)_2_SO_4_, 0.20 g kg^-1^ P as NaH_2_PO_4_ and 0.30 g kg^-1^ K as KCl. Seedlings of *S. plumbizincicola* were obtained from a heavy-metal polluted area in Zhejiang Province, east China and were ∼5 cm long with a pair of leaves and 4–5 nodes. Healthy plants of uniform size were chosen for the pot experiment after the seedlings had produced roots under incubation in half-strength Hoagland nutrient solution for 2 weeks.

**Table 1:** Selected physicochemical properties of the soil used in the pot experiment.

Soil property	Value
pH (H_2_O)	4.7
Organic matter (g kg^-1^)	32.0
Total N (g kg^-1^)	2.2
Total P (g kg^-1^)	0.5
Total K (g kg^-1^)	11.2
Hydrolyzable nitrogen (mg kg^-1^)	91.0
Available P (mg kg^-1^)	8.6
Available K (mg kg^-1^)	55.0
Cd (mg kg^-1^)	0.53

In the pot experiment five treatments were set up in a fully randomized layout, namely: (1) uninoculated *S. plumbizincicola* as control (CK); (2) *S. plumbizincicola* inoculated with solid fermentation powder of *T. reesei* FS10-C (SP); (3) *S. plumbizincicola* inoculated with sterilized solid fermentation powder (SCK); (4) *S. plumbizincicola* inoculated with conidium wettable powder of *T. reesei* FS10-C (WP); (5) *S. plumbizincicola* inoculated with conidium wettable powder without the addition of *T. reesei* FS10-C conidia (WCK). The inoculation method for the SP and SCP treatments was hole fertilization (4% inoculum was added), whereas spray irrigation (100 mL per pot was added at 0, 30, 60, 90, and 120 days respectively) was used for the WP and WCP treatments. Each treatment was set up in quadruplicate, giving a total of 20 pots. Each pot received 1.5 kg soil and five plants.

The plants grew in a growth chamber under controlled light (14-h photoperiod at 1.5 × 10^4^ lux), temperature (25/20°C, light/dark), and humidity (60–70%). Throughout the experiment the plants were watered with deionized water to maintain 70% of water-holding capacity. Shoot and soil samples were collected after incubation for 120 days. Shoot fresh and oven dry weights (DWs) were determined. A portion of the soil samples was air-dried, ground and sieved (0.15 mm) before analysis for pH and available phosphorus (P). Soil pH was measured using a pH meter (520M-01, Thermo Orion, Beverly, MA, USA) and soil available P was determined based on the Olsen method (Olsen et al., 1954). The remaining potion of the soil samples was sieved (<2 mm) for subsequent experiments.

### Analysis of Cd in Soil and Plant Samples

Ground plant samples weighing 0.5 g were placed into digestion vials and mixed with 10 mL of HClO_4_:HNO_3_ (2:3 v/v). Dry soil samples (0.25 g) were weighed and mixed with 14 mL of HCl:HNO_3_ (4:1 v/v). All samples were digested according to the EPA Method 3050B (EPA, 1996) using the Hot Block Digestion System (SISP, DS-360). After digestion all samples were cooled completely and then diluted to 50 mL. Cd concentrations were measured using atomic absorption spectrophotometry (Varian SpectrAA 220Z). In addition, blank and certified reference materials (GSV-2 for plant analysis, GSS-4 for soil analysis, Chinese geological reference materials) were used for quality control.

### Soil Enzyme Activities

Soil dehydrogenase (DHA) activity was assessed by a modification of the method of Singh and Singh (2005). Sieved soil (5 g) was weighed and mixed with 5 mL of 0.5% 2, 3, 5-triphenyltetrazolium chloride (TTC) solution and incubated for 12 h at 30°C in the dark. After incubation triphenylformazan (TPF), formed by the reduction of TTC, was extracted with three batches of 100 mL methanol, shaken at 300 rpm for 1 h and centrifuged at 2,000 rpm for 5 min. The supernatant was filtered and the concentration of TPF was determined spectrophotometrically at 485 nm. Blanks with TTC omitted were included. All results are expressed as μg g^-1^ dw.

Fluorescein diacetate (FDA) hydrolysis activity was determined according to Aira and Domínguez (2014). Briefly, 5 g of sieved soil was incubated for 20 min at 30°C and 200 rpm with 15 mL of 60 mM potassium dihydrogen phosphate buffer (pH 7.6) and 0.2 mL of FDA stock solution (1000 μg mL^-1^). The reaction was stopped by adding 15 mL of chloroform/methanol (1:1 v/v) and the mixture was gently mixed and centrifuged at 2,000 rpm for 3 min. The supernatant was filtered and read at 490 nm. The results are expressed as μg g^-1^ dw.

### Soil Microbial Biomass C

The fumigation-extraction method (Vance et al., 1987) was used to determine soil microbial biomass C according to Beck et al. (1997). Ten grams of sieved soil for chloroform-fumigated and non-fumigated treatments were extracted with 50 mL of 0.5 mol L^-1^ K_2_SO_4_ and then filtered at 300 rev min^-1^ for 30 min. Organic C in the supernatant was measured using an automated TOC Analyzer. Microbial biomass C was calculated as follows: biomass C = Ec/k_EC_, where Ec = (organic C extracted from fumigated soil) - (organic C extracted from non-fumigated soil), and k_EC_, which was used to convert the measured flush of C to biomass C, was 0.45 (Beck et al., 1997; Yao et al., 2003).

### Biolog^®^ EcoPlate Analysis of the Soil Microbial Community

Soil bacterial functional diversity was assessed as described by Yao et al. (2003). Briefly, 10 g of sieved soil was added to 100 mL of sterile water in a 250-mL flask and shaken at 180 rpm for 10 min. Ten-fold serial dilutions were made and 150 μL of the final 10^-3^ dilution was added to each well of a Biolog^®^ EcoPlate. The plates were incubated at 28°C for 7 days and color development in each well was recorded as the absorbance at 590 nm using a microplate reader (BioTek μQuant, Winooski, VT, USA) at regular 12-h intervals. Microbial metabolic activity in each microplate, expressed as the average well-color development (AWCD), was determined as follows: AWCD = Σ ODi/31, where ODi is the optical density value from each well (Fang et al., 2009). The McIntosh index (U) was calculated as $U\;=\sqrt{\left(\sum {\mathrm{ni}}^{2}\right)}$, where ni refers to the absorbance value (McIntosh, 1967). The Shannon–Weaver index (H) was calculated as H = –Σ Pi (ln Pi), where Pi is the ratio of the activity of each substrate (ODi) to the sum of activities of all substrates (Σ ODi; Zhong et al., 2009).

### Quantitative Real-Time PCR Assay

The abundance of *Trichoderma* sp. was quantified by real-time PCR (Bio-Rad, Hercules, CA, USA) performed on a Real-Time System and 18S rDNA amplifications were performed in a total volume of 20 μL containing 2 μL of soil microbial DNA, 0.2 μL of each of the primers DG (5′-CTGGCATCGATGAAGAACG-3′) and DT (5′-ATGCGAGTGTGCAAACTACTG-3′) and 10 μL of SYBR Green I. The qPCR was performed with an initial denaturation and enzyme activation step for 5 s at 95°C, followed by 40 cycles of 30 s at 95°C, 30 s at 53°C, and 30 s at 72°C and a final extension performed at 72°C for 5 min. Fluorescence measurements were made at the end of each annealing cycle and an additional measuring point at 80°C for 1 s to detect the formation of primer dimers during amplification. A melt curve analysis was performed by raising the temperature from 65 to 95°C in 0.2°C steps for 1 s each. The results are expressed as copies g^-1^ dw.

### Data Analysis

To evaluate the phytoremediation effects of *S. plumbizincicola* we defined phytoextraction efficiency (%) as:

$$\frac{The Cd content accumulated in the plant shoots after phytoremediation}{The initial soil Cd content}$$

and the removal efficiency (%) as:

$$\frac{\begin{array}{c} The difference of Cd content in the soil between the\\ beginning and end of phytoremediation \end{array}}{The initial soil Cd content}.$$

The pot experiment was performed in quadruplicate and the other experiments in triplicate. All results are presented as the mean ± SD. Data processing and correlation analysis were performed using MS Excel 2003. The results related to Cd phytoremediation and soil properties were analyzed by one-way analysis of variance using SigmaPlot 12.5 and all pairwise multiple comparison analyses (Holm-Sidak method) were performed at the *p* < 0.05 level.

## Results and Discussion

### Cadmium tolerance of *T. reesei* FS10-C

**FIGURE 1:**
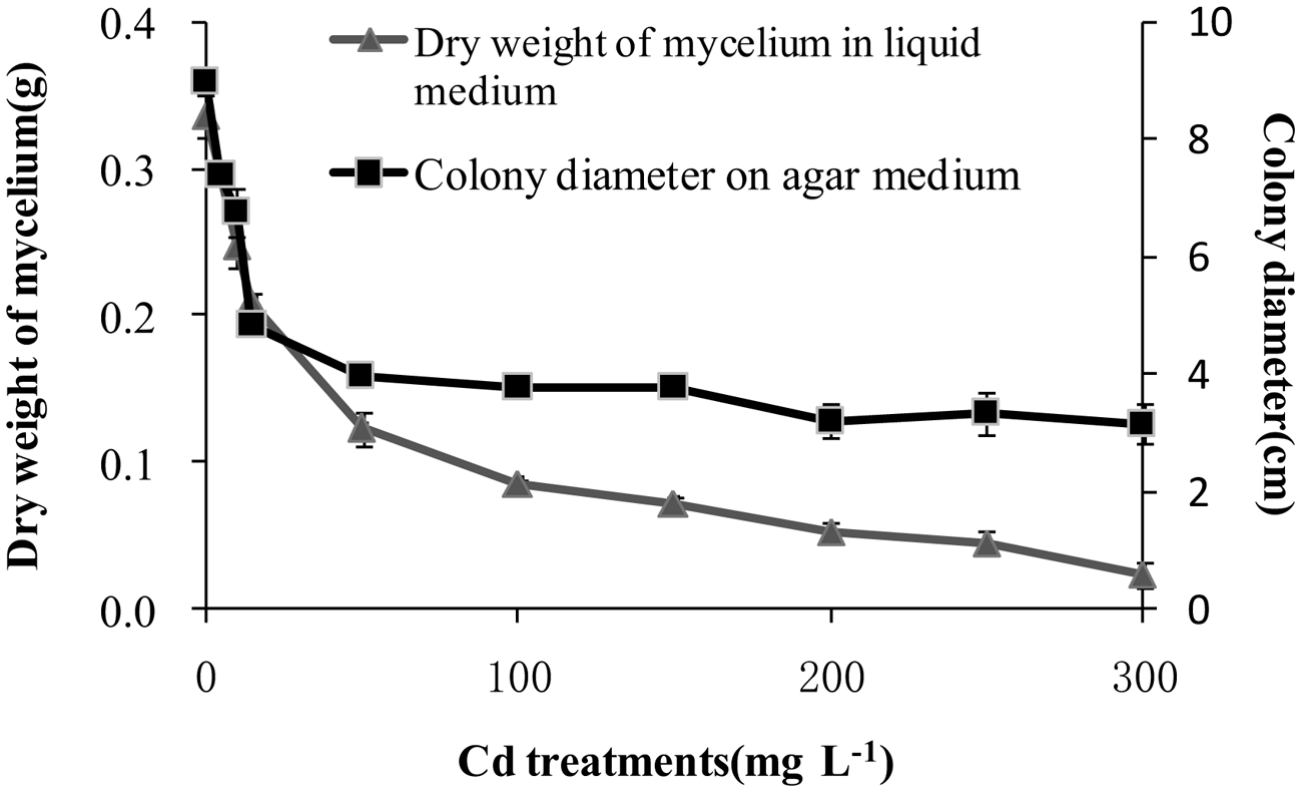
**Effect of Cd on the growth of *Trichoderma reesei* FS10-C.** Values are mean ± SD of triplicate determinations.

**FIGURE 2:**
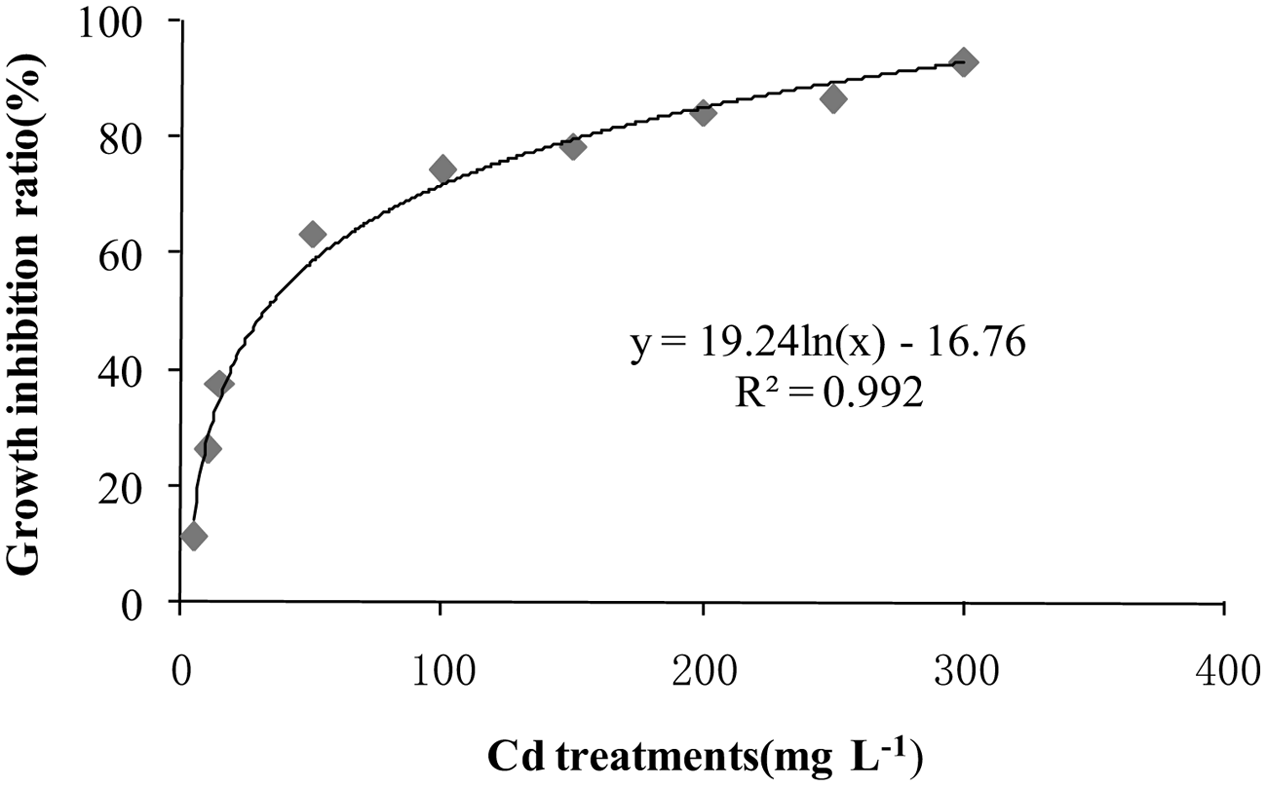
**The growth inhibition ratio of *T. reesei* FS10-C in liquid medium**.

After incubation at 28°C for 7 days the growth of *T. reesei* FS10-C on solid and in liquid media with different Cd treatments exhibited similar trends (**Figure 1**). As the Cd concentration increased, *T. reesei* FS10-C experienced more pronounced growth inhibition. However, FS10-C was still able to grow at a concentration of 300 mg L^-1^ Cd^2+^. The growth inhibition ratio of FS10-C is shown in **Figure 2** and indicates that FS10-C tolerated high Cd stress.

**FIGURE 3:**
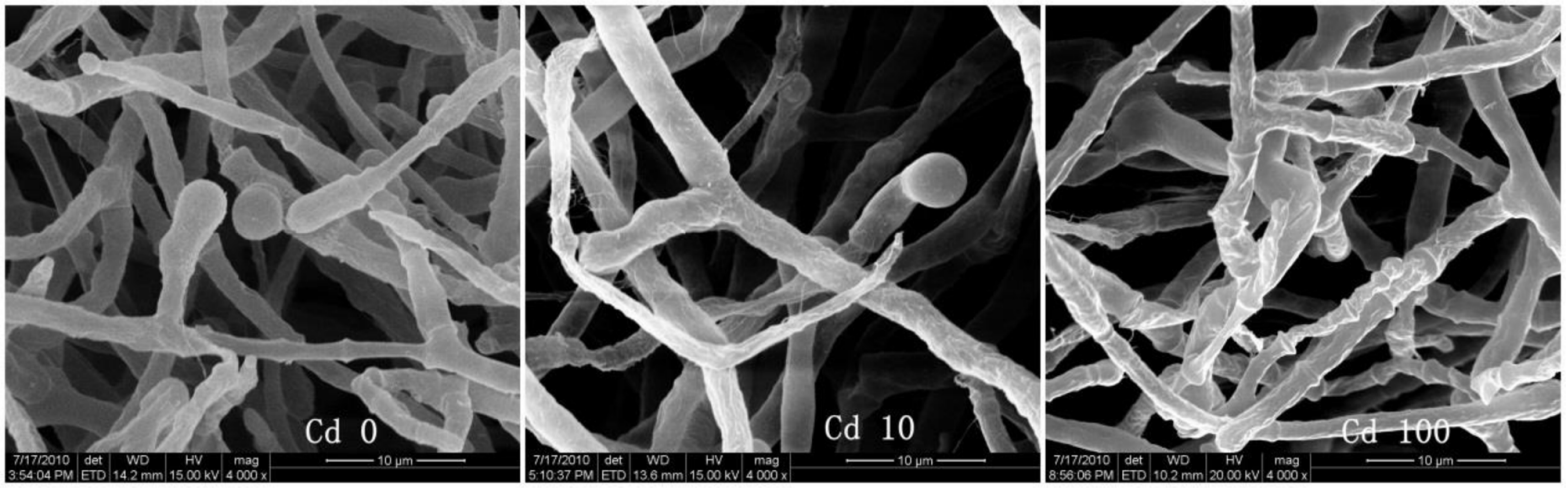
**Effect of Cd on the morphology of *T. reesei* FS10-C**.

**FIGURE 4:**
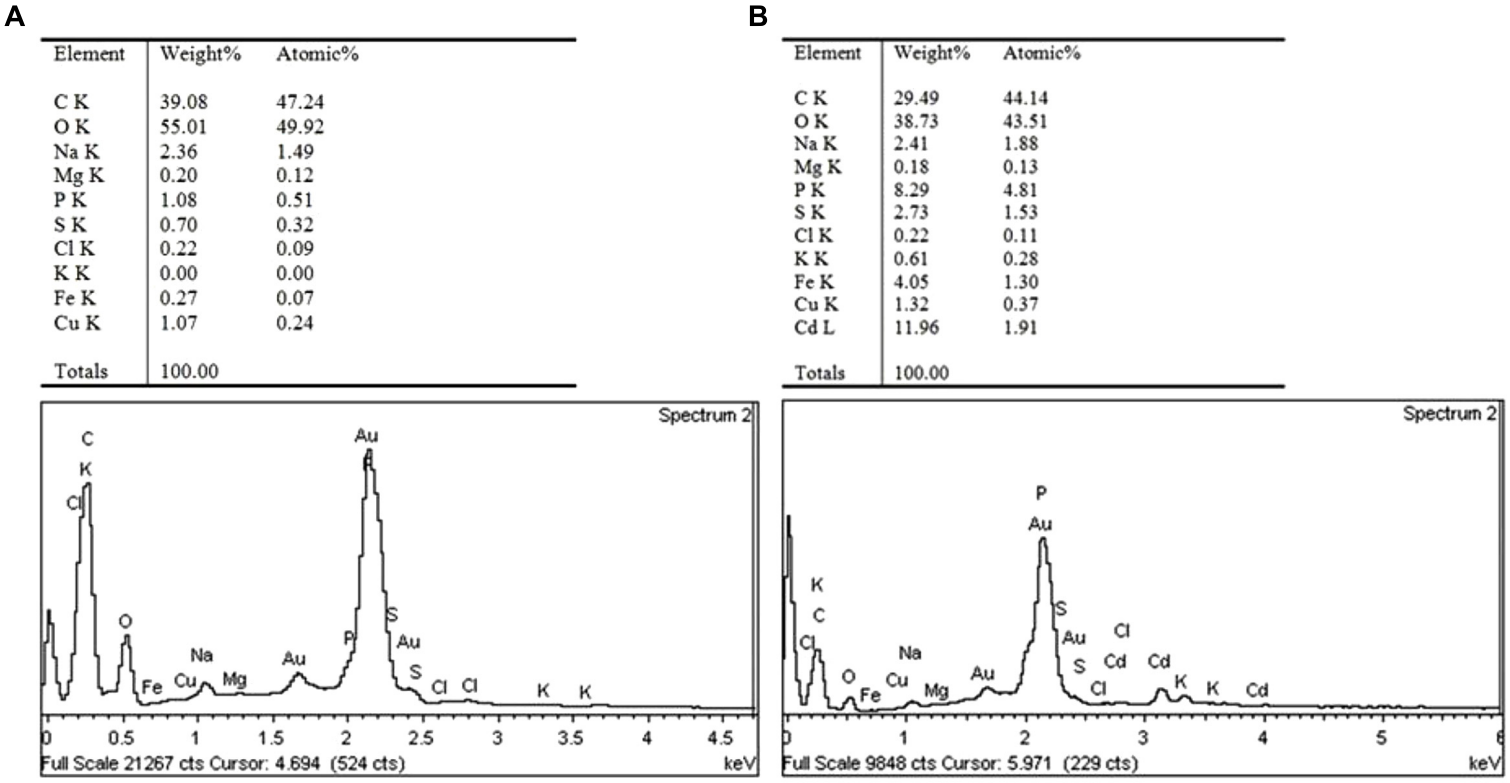
**The elemental contents of mycelia under **(A)** 0 mg Cd L^-1^ and **(B)** 100 mg Cd L^-1^ treatments**.

To better understand the Cd tolerance of FS10-C its morphological changes under different Cd treatments were investigated using SEM (**Figure 3**). The results suggested that there was no significant influence on FS10-C growth in the 10 mg L^-1^ Cd treatment compared with the control, although a small irregular fold emerged on the surface of FS10-C mycelia in the 100 mg L^-1^ Cd treatment. Furthermore, EDX analysis showed that the peaks of the four nutrient elements P, S, K, and Fe increased (**Figure 4**).

Cao et al. (2008) found that *T. atroviride* F6 resisted up to 100 mg Cd^2+^ L^-1^ in liquid media. Sahu et al. (2012) indicated that the DW of *T. viride* mycelia was 0.07 g in 200 ppm Cd. In addition, Babu et al. (2014a) showed that *Trichoderma* sp. PDR1-7 survived in 100 mg L^-1^ Cd with 0.9 g dw. Our results showed that *T. reesei* FS10-C survived in 300 mg L^-1^ Cd in both solid and liquid media, indicating that *T. reesei* FS10-C has a high level of Cd tolerance.

### Increasing Growth and Cd Uptake by *Sedum plumbizincicola* When Inoculated with *T. reesei* FS10-C

Shoot biomass after 120 days is shown in **Table 2**. The shoot biomass values under the four treatments using inoculation agents were all higher than the control in the following declining sequence: SP > SCK > WP> WCK > CK. Compared with the control, the fresh shoot weights in the SP and SCK treatments were enhanced by 53 and 30%, respectively (*p* < 0.05), and their corresponding DWs were enhanced by 61 and 35% (*p* < 0.05). Shoot DW under the WP treatment (12.81 g pot^-1^) was also significantly higher (*p* < 0.05) than the control (10.20 g pot^-1^) but there was no significant increase in fresh shoot weight under the WP and WCK treatments or shoot DW in the WCK treatment.

**Table 2:** Effect of inoculation agents on shoot biomass and Cd uptake of *Sedum plumbizincicola* and phytoremediation.

Treatmen*t*	Fresh weight (g pot^-1^)	Dry weight (g pot^-1^)	Cd uptake (mg pot^-1^)	Cd conc. in soil (mg kg^-1^)	Phytoextraction efficiency (%)	Removal efficiency (%)
CK	123.79 ± 4.39^c^	10.20 ± 0.15^c^	0.30 ± 0.04^b^	0.27 ± 0.02^b^	37.24 ± 4.51^b^	49.45 ± 4.43^b^
SCK	160.76 ± 16.39^ab^	13.73 ± 0.97^b^	0.37 ± 0.05^ab^	0.24 ± 0.01^ab^	47.12 ± 6.83^ab^	54.27 ± 1.94^ab^
SP	189.25 ± 23.35^a^	16.44 ± 1.86^a^	0.46 ± 0.05^a^	0.21 ± 0.01^a^	58.30 ± 6.37^a^	60.50 ± 1.24^a^
WCK	131.50 ± 4.73^bc^	11.79 ± 0.59^bc^	0.33 ± 0.06^b^	0.24 ± 0.03^ab^	41.02 ± 7.30^b^	54.40 ± 5.73^ab^
WP	141.98 ± 12.10^bc^	12.56 ± 0.73^b^	0.44 ± 0.03^a^	0.22 ± 0.01^a^	54.84 ± 3.48^a^	58.56 ± 2.39^a^

Mean values (±SD) followed by different letters with in a column differ significantly at *p* < 0.05.

Shoot Cd uptake is also shown in **Table 2**. Cadmium accumulation under the SP and WP treatments was 0.46 mg pot^-1^ and 0.44 mg pot^-1^ respectively and these values were significantly higher (*p* < 0.05) than the control treatment (0.30 mg pot^-1^). Cadmium uptake under the SCK and WCK treatments did not increase significantly over the control. A significant decline (*p* < 0.05) was observed in all treatments (including the control) compared with the initial soil Cd content, perhaps attributable to the Cd accumulation potential of *S. plumbizincicola.* The phytoextraction and removal efficiencies of *S. plumbizincicola* alone (the control treatment) were 37.2 and 49.5%, respectively (**Table 2**). After the addition of inoculation agents the phytoextraction efficiency of *S. plumbizincicola* increased by 41.0–58.3% and the removal efficiency increased by 54.3–60.5% (**Table 2**). In addition, SP and WP treatments with *T. reesei* FS10-C showed greater phytoextraction capabilities and removal efficiencies than SCK and WCK treatments without FS10-C.

Our results showed that the declining sequence of shoot biomass among all treatments was: SP > SCK > WP > WCK > CK. It can be concluded that the use of solid fermentation powder as an inoculation agent significantly enhanced (*p* < 0.05) the growth of *S. plumbizincicola* with a distinct advantage over conidium wettable powder. In addition, SP > SCK and WP > WCK showed that the inoculation agents with *T. reesei* FS10-C were more effective than inoculation agents without FS10-C, indicating that FS10-C played an important role in promoting the growth of *S. plumbizincicola*. Higher plant biomass under the SCK treatment as compared to the control indicates possible effects of nutrients and *Trichoderma* secondary metabolites in the inoculation agent for SCK promoting plant growth (Hoyos-Carvajal et al., 2009; Vinale et al., 2009). The sequence of shoot Cd uptake was: SP > WP > SCK > WCK > CK. Cadmium uptake in the SP and WP treatments was significantly higher than the SCK and WCK treatments (*p* < 0.05), indicating that the inoculation agents with *T. reesei* FS10-C were more effective in enhancing Cd uptake by *S. plumbizincicola* than those inoculation agents without FS10-C. In addition, the sequence SP > WP and SCK > WCK showed that higher plant biomass accumulated more Cd.

In general, our study demonstrated that *T. reesei* FS10-C was able to enhance the plant biomass and Cd accumulation of *S. plumbizincicola*, especially with an inoculation agent such as solid fermentation powder of FS10-C. Certain other *Trichoderma* sp. have also been reported to enhance plant growth and Cd uptake under Cd stress. Adams et al. (2007) showed that inoculation with *T. harzianum* Rifai 1295-22 increased the DW of crack willow and Cd accumulation in the shoots by 39 and 24%, respectively. Babu et al. (2014b) found that inoculation with *T. virens* PDR-28 increased the DW of maize shoots and Cd accumulation by 56 and 59%. In our study inoculation with solid fermentation powder of FS10-C increased the DW of *S. plumbizincicola* shoots by 61% and shoot Cd accumulation by 53%, representing an enhancement of Cd phytoremediation. Plant growth promotion by inoculation with FS10-C was mainly attributable to the production of IAA, ACC deaminase and siderophores as well as phosphate solubilization (Gravel et al., 2007; Qi and Zhao, 2013). Qualitative and quantitative analyses of plant growth-promoting traits of FS10-C (Triveni et al., 2013; Kotasthane et al., 2015) have been carried out. The results showed that FS10-C had the ability to produce ACC deaminase and siderophores and to solubilize phosphate (Supplementary Figures S1 and S2; average siderophore production was 68%) and this agrees with the findings of Babu et al. (2014b,c). Increased Cd uptake induced by FS10-C might be attributed to the successful colonization of FS10-C both promoting plant growth to accumulate more Cd and enhancing Cd phytoextraction by altering the solubility, availability and transport of Cd (Song et al., 2015).

### Changes in Soil pH and Available P

After 120 days there was a clear decline (*p* < 0.05) in soil available P concentration in all treatments compared with the initial value (8.6 mg kg^-1^), but there was no significant difference in soil pH (**Table 3**). Compared with the control the pH under the SP treatment increased significantly (*p* < 0.05). Soil available P under the SP and SCK treatments increased substantially (*p* < 0.05) by 107 and 71%, respectively, compared with the control.

**Table 3:** Soil pH and available P concentration after phytoremediation for 120 days.

Treatment	pH	Available P (mg kg^-1^)
CK	4.63 ± 0.03^bc^	3.60 ± 0.11^c^
SCK	4.84 ± 0.14^a^	6.15 ± 0.46^b^
SP	4.80 ± 0.06^ab^	7.43 ± 0.40^a^
WCK	4.68 ± 0.09^ac^	2.64 ± 0.19^d^
WP	4.55 ± 0.06^c^	3.25 ± 0.09^c^

Mean values (±SD) followed by different letters with in a column differ significantly at *p* < 0.05.

Soil available P under both SP and SCK treatments was significantly higher than the control (*p* < 0.05), indicating that *T. reesei* FS10-C played important role in enhancing nutrient release into the soil. Furthermore, more soil available P under the SP treatment than SCK showed that the role of FS10-C was more prominent. This was likely due to the phosphate solubilizing capacity of FS10-C. However, the soil available P values under both WP and WCK treatments were lower than the control. This might be attributable to the spore content of FS10-C being quite small in the inoculation agent of the WP treatment and the inoculation agents in both WP and WCP treatments being added by foliar spray which would have made little contribution to the solubilization of P in the soil. The overall decline in soil available P under all treatments compared with the initial value might be attributable to plant nutrient uptake as available P was positively related to plant fresh and DWs with *R*^2^ values of 0.86 and 0.72, respectively. In addition, our results showed that soil pH values also increased under both SP and SCK treatments in addition to soil available P, which differed with the earlier conclusion that soil available P was negatively related to soil pH (Deng et al., 2012; Babu et al., 2014c). This was likely due to the soil pH value being neutralized after the addition of the inoculation agent.

### Enhancement of Soil Microbial Activities by Inoculation Agents

Microbial biomass C, DHA and FDA hydrolysis activities under the four treatments with the addition of inoculation agents were enhanced to different degrees after phytoremediation (**Table 4**). The highest increase in soil microbial biomass C (145%, *p* < 0.05) was observed under the SP treatment, followed by SCK and WP treatments with increases of 62 and 62%, respectively (*p* < 0.05). Similarly, the highest DHA activity was found under the SP treatment, followed by SCK, WP and WCK, and all exhibited significantly higher levels than the control with increases of 86, 56, 49, and 20% (*p* < 0.05), respectively. In terms of FDA hydrolysis activity, the four treatments with the addition of inoculation agents were significantly higher than the control, with increases of 69, 69, 49, and 34% (*p* < 0.05). Furthermore, the treatments involving inoculation with conidium wettable powder with or without *T. reesei* FS10-C clearly demonstrated greater ability to increase FDA hydrolysis activity than those inoculated with solid fermentation powder with or without *T. reesei* FS10-C.

**Table 4:** Microbial biomass C and soil enzyme activities after phytoremediation for 120 days.

Treatment	Microbial biomass C μg g^-1^	DHA activity μg g^-1^ dw	FDA hydrolysis activity μg g^-1^ dw × 20 min
CK	217.21 ± 48.96^c^	55.82 ± 3.05^d^	17.60 ± 2.78^c^
SCK	340.15 ± 44.69^b^	86.67 ± 4.05^b^	23.18 ± 0.39^b^
SP	522.51 ± 73.52^a^	103.80 ± 5.23^a^	25.83 ± 3.14^b^
WCK	246.08 ± 12.20^c^	66.98 ± 2.07^c^	29.27 ± 2.04^a^
WP	341.71 ± 26.26^b^	82.71 ± 1.97^b^	29.28 ± 1.36^a^

Mean values (±SD) followed by different letters with in a column differ significantly at *p*< 0.05.

The maintenance of soil fertility depends on the microbial biomass and its activities which are of primary importance in nutrient cycling and ecosystem sustainability, and microbial biomass and activities are sensitive to changes in soil HM content (Giller et al., 1998). One of the soil microbiological parameters, microbial biomass C, is considered to be a sensitive indicator of HM toxicity and soil quality (Yao et al., 2003). Our data suggested that all inoculation agents with or without *T. reesei* FS10-C, particularly the solid fermentation powder of *T. reesei* FS10-C, increased microbial biomass C. In addition, soil DHA activity based on the metabolic state of the soil biota (Araujo et al., 2015) is also used to assess soil health (Gil-Sotres et al., 2005). Our results showed that all inoculation agents tested enhanced DHA activity, indicating that the general activities of soil microbes were enhanced. FDA hydrolysis activity is also a good indicator of soil health in the presence of metal contaminants (Frølund et al., 1995; Gil-Sotres et al., 2005). All inoculation agents increased FDA hydrolysis activity, indicating that the metabolism of soil microbes was promoted, thus resulting in the enhanced microbial activities.

### Changes in Microbial Community Functional Diversity after Phytoremediation

**FIGURE 5:**
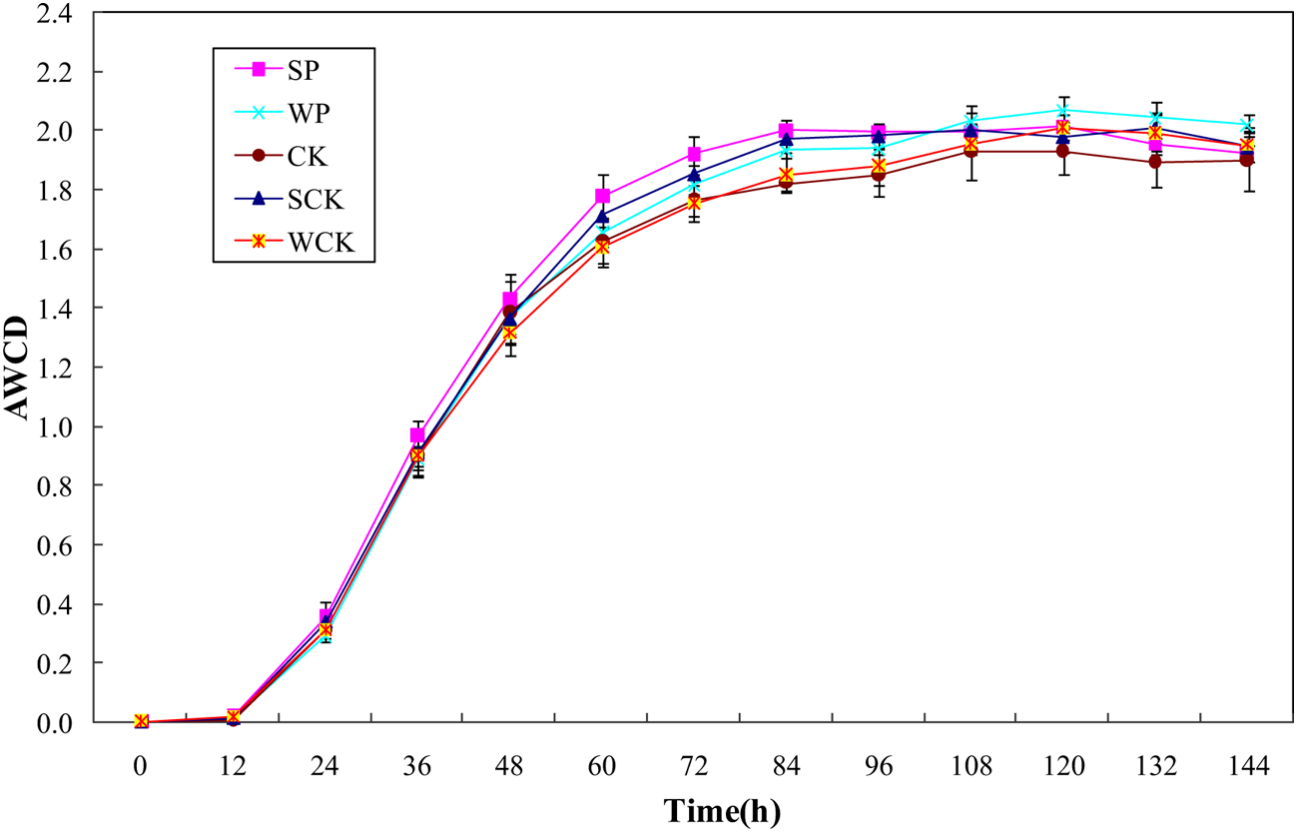
**Average well color development (AWCD) values of microbial communities under the different treatments.** Values are means ± SD of triplicate measurements.

Changes in AWCD reflecting the oxidative catabolism of the microbial community over time are shown in **Figure 5**. It is clear that AWCD values for all treatments initially increased rapidly until 84 h and then tended to slow down. The average AWCD values for SP, SCK, WP, and WCK treatments were 2.00, 1.97, 1.93, and 1.85 respectively. SP, SCK and WP treatments were significantly higher than the control (1.82, *p* < 0.05) but WCK treatment did not significantly increase compared with the control. The Shannon–Weaver index and McIntosh index are used to indicate microbial community richness (Fang et al., 2009) and evenness (McIntosh, 1967), respectively. The data in **Table 5** indicate that there was no significant difference between the values of Shannon–Weaver index under all treatments. The values of the McIntosh index under all treatments reached a level of 10.68–11.61 (**Table 5**) and the values under SP, SCK, and WP treatments were significantly higher than that in the control (*p* < 0.05).

**Table 5:** Shannon–Weaver index (H) and McIntosh index (U) based on community level physiological profiles detected using a Biolog EcoPlate.

Treatment	H (84 h)	U
CK	3.371 ± 0.021^a^	10.681 ± 0.054^b^
SCK	3.381 ± 0.014^a^	11.435 ± 0.243^a^
SP	3.374 ± 0.009^a^	11.607 ± 0.519^a^
WCK	3.364 ± 0.012^a^	10.822 ± 0.259^b^
WP	3.362 ± 0.024^a^	11.310 ± 0.262^a^

Mean values (± SD) followed by different letters within a column differ significantly at *p* < 0.05.

Microbial community structure is also considered to be a biological indicator of HM stress (Doelman et al., 1994). The Biolog^®^ EcoPlate method combined with other analyses such as the diversity indices has been recommended to evaluate whole soil microbial community functional diversity (Ros et al., 2008). Our data showed that the evenness of the soil microbial community was enhanced by all inoculation agents but no significant variation in richness. It was likely due to the low levels of Cd contamination in the soil and the similar experimental conditions in each of the treatments. A similar study also indicated that differences in flora could not be distinguished sensitively by the diversity indices when the experimental conditions did not differ significantly between different treatments (Drenovsky et al., 2008). In addition, the AWCD values were also enhanced by all inoculation agents and found to be closely correlated with microbial biomass C (*R*^2^ = 0.81), indicating that microbial biomass and metabolic activity were both enhanced by the addition of the inoculation agents. High concentrations of HMs affect the functional diversity of soil microbial community, resulting in a decline in soil quality (Chander and Brookes, 1993). Our results demonstrated that the soil microbial community might be protected and even enhanced by phytoremediation using *S. plumbizincicola* with all tested inoculation agents, particularly the solid fermentation powder of *T. reesei* FS10-C.

### *Trichoderma* Colonization Ability of Solid Fermentation Powder of *T. reesei* FS10-C

**FIGURE 6:**
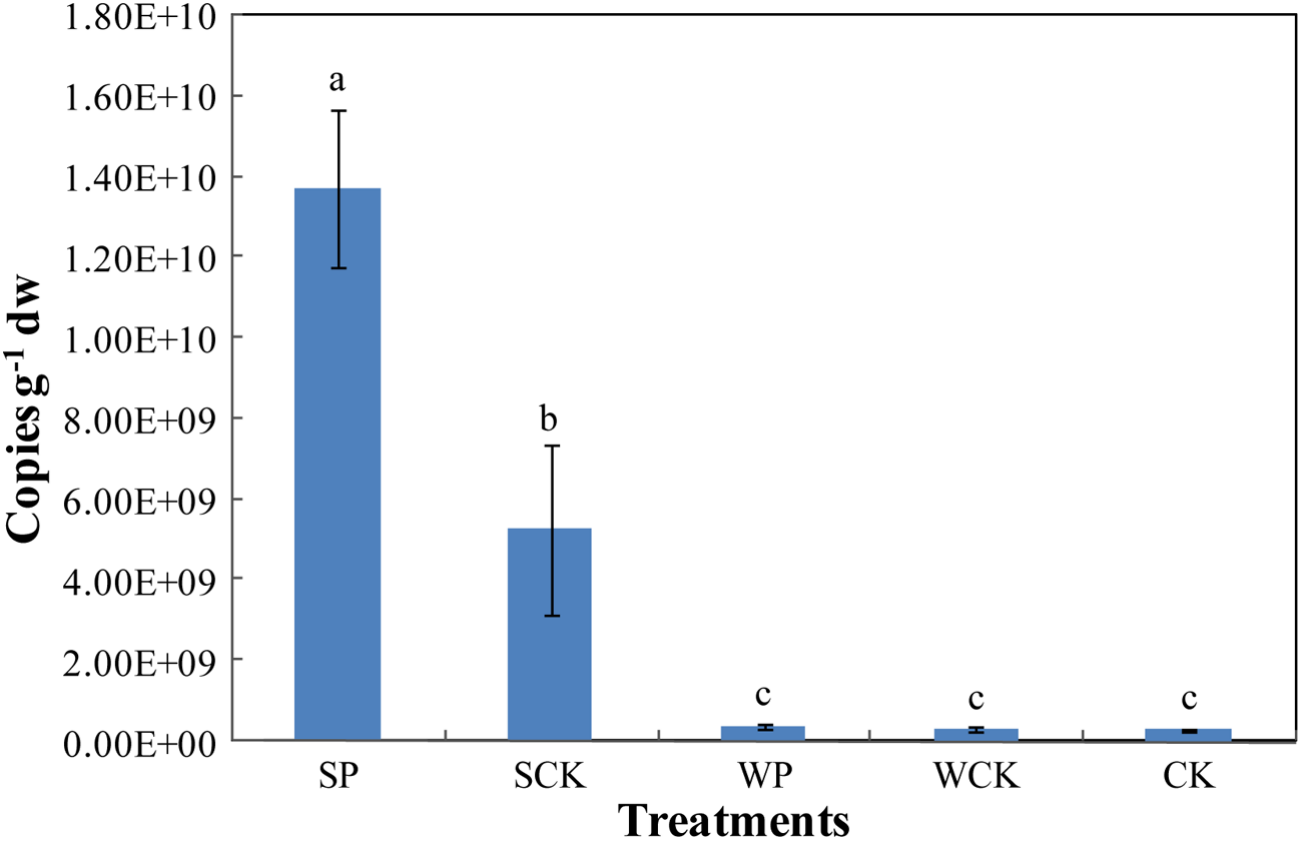
**The gene copy numbers of soil *Trichoderma* sp. under the different treatments.** Values are means ± SD of triplicate measurements.

The abundance of *Trichoderma* sp. was estimated via the real-time quantification of its rDNA gene copy numbers (**Figure 6**). The results show that the abundance of *Trichoderma* sp. under SP treatment reached its highest level of 1.37 × 10^10^ copies g^-1^ dw after phytoremediation, followed by 5.26 × 10^9^ copies g^-1^ dw under the SCK treatment. Both were significantly higher (*p* < 0.05) than the other three treatments (WP: 3.94 × 10^8^ copies g^-1^ dw, WCK: 2.92 × 10^8^ copies g^-1^ dw, CK: 2.98 × 10^8^ copies g^-1^ dw), which were not significantly different from each other.

It is clear that the SCK treatment resulted in a high *Trichoderma* gene abundance, suggesting that the organic substance present in the inoculation agent supplied nutrients and promoted the growth of indigenous *Trichoderma* in the soil. In addition, the *Trichoderma* gene abundance under the SP treatment was significantly higher (*p* < 0.05) than SCK, indicating that FS10-C helped *Trichoderma* sp. to be the most advantaged flora in the soil and enhanced the colonization ability of *Trichoderma*. The *Trichoderma* gene copies were low in the WP and WCK treatments. Possible explanations for this are restriction of the formation of advantageous flora by the decreased FS10-C spore content and the addition of the two inoculation agents (foliar spray) being adverse to the rhizosphere gene expression of FS10-C.

Babu et al. (2014b) indicated that plant growth and HM uptake enhancement were attributable to the successful colonization of *T. virens* PDR-28 in soil. Our results showed that *Trichoderma* gene abundance was positively related to plant DW, Cd uptake, available P, microbial biomass C and DHA activity (*R*^2^ values were 0.84, 0.41, 0.88, 0.85, and 0.72, respectively). Thus, we supposed that the successful colonization of *T. reesei* FS10-C was contributed to the enhancement of plant growth, Cd uptake, nutrient release, microbial biomass and activities. This also indicated that *T. reesei* FS10-C was a good candidate for the enhancement of Cd phytoremediation.

## Conclusion

To our knowledge this is the first report demonstrating the potential of *T. reesei* FS10-C to promote plant growth and the Cd removal capacity of *S. plumbizincicola* grown in Cd-contaminated soil. Our results showed that all the inoculation agents tested were able to increase plant biomass and Cd uptake with the simultaneous inoculation of FS10-C compared with inoculation without FS10-C thereby increasing the efficiency of phytoremediation by *S. plumbizincicola*. In addition, nutrition release and microbial activities were promoted, particularly by the solid fermentation powder of FS10-C, indicating an enhancement of soil fertility after phytoremediation. We also found that *Trichoderma* colonization ability played an important role in enhancing plant growth, Cd removal and soil fertility. Solid fermentation powder of *T. reesei* FS10-C showed the greatest *Trichoderma* colonization ability and thus the greatest potential for use as an inoculation agent to enhance Cd phytoremediation. However, field experiments need to be conducted to further verify the practical effects of the application of solid fermentation powder of FS10-C under field conditions.

## Conflict of Interest Statement

The Guest Associate Editor Ying Ma declares that, despite having collaborated with the authors Ying Teng, Yongming Luo and Zhengao Li, the review process was handled objectively. The authors declare that the research was conducted in the absence of any commercial or financial relationships that could be construed as a potential conflict of interest.
