# Supplementary material for: Trichoderma reesei FS10-C enhances phytoremediation of Cd-contaminated soil by Sedum plumbizincicola and associated soil microbial activities
Source: Front Plant Sci. 2015 Jun 10;9:220. doi: 10.3389/fpls.2015.00438 (PMC4461814; doi:10.3389/fpls.2015.00438)
Supplement: Supplementary file 1 [file Presentation_1.PDF]

Supplementary Material

Trichoderma reesei FS10-C enhances phytoremediation by *Sedum plumbizincicola* for Cd-contaminated soils and associated soil microbial activities

Teng Ying[a*], Luo Yang[a], Ma Wenting[a], Zhu Lingjia[a], Ren Wenjie[a], Luo Yongming[a,b], Li Zhengao[a]

a Key Laboratory of Soil Environment and Pollution Remediation, Institute of Soil Science, Chinese Academy of Sciences, Nanjing 210008, China

b Yantai Institute of Coastal Zone Research, Chinese Academy of Sciences, Yantai 264003, China

***Correspondence:**

Ying Teng, yteng@issas. ac.cn
Key Laboratory of Soil Environment and Pollution Remediation Institute of Soil Science, Chinese Academy of Sciences, East Beijing Road No. 71, Nanjing, Jiangsu Province, 210008, China

This file includes:

Supplementary Figures S1 and S2

Supplementary Figures

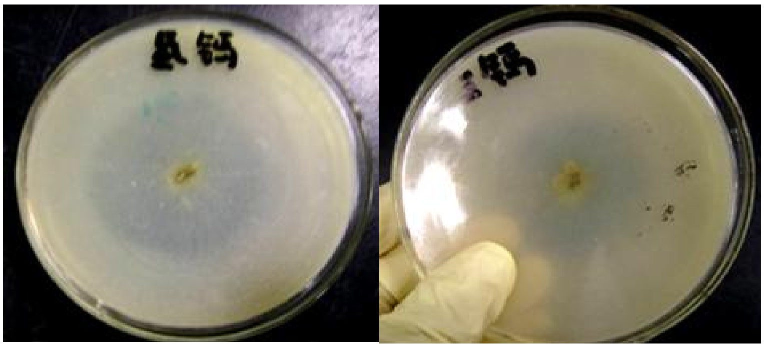

(a) The plate with $CaHPO_4$ (b) The plate with $Ca_3(PO_4)_2$

Fig. S1 Inorganic phosphate solubilization ability of *T. reesei* FS10-C. The circles of dissolved P were 54.2 ± 0.3 mm (a) and 45.2 ± 0.2 mm (b).

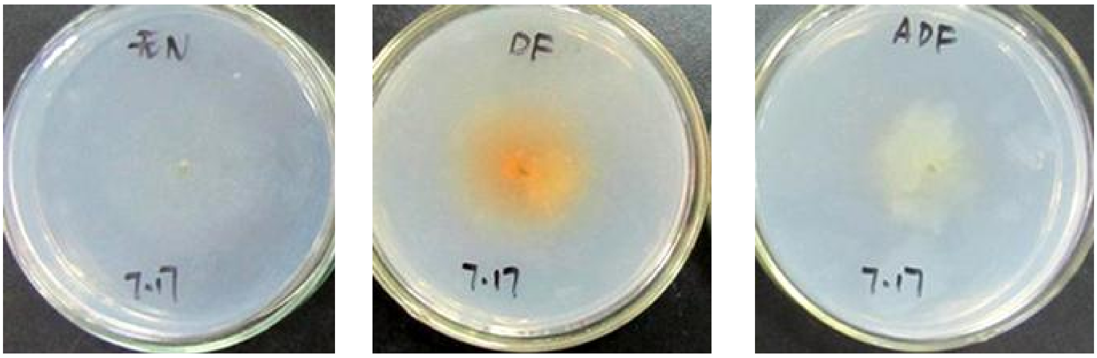

(a) Nitrogen free medium (b) DF medium (c) ADF medium

Fig. S2 ACC deaminase production ability of *T. reesei* FS10-C. The DF medium (pH 7.5) was composed of 4.0 g L^{-1} KH_2PO_4, 6.0 g L^{-1} Na_2HPO_4, 0.2 g L^{-1} $MgSO_4{\cdot}7H_2O$, 2.0 g L^{-1} glucose, 2.0 g L^{-1} gluconic acid, 2.0 g L^{-1} citric acid, 2.0 g L^{-1} $(NH_4)_2SO_4$, 0.1 mL $FeSO_4{\cdot}7H_2O$, 0.1 mL microelement solution and 18 g L^{-1} agar. The ADF medium was prepared using 3.0 mmol L^{-1} ACC to replace 2.0 g L^{-1} $(NH_4)_2SO_4$. The DF medium without the addition of $(NH_{42})SO_4$ was used as the nitrogen free medium.
